# Antimicrobial Effects of Egyptian Local Chicory, *Cichorium endivia* subsp*. pumilum*

**DOI:** 10.1155/2018/6475072

**Published:** 2018-11-04

**Authors:** Ahmed M. Amer

**Affiliations:** Department of Plant Biotechnology, National Research Centre (NRC), Cairo 12622, Egypt

## Abstract

The discovery of novel and more efficient antimicrobial agents from natural sources like plants is one of the most important ways through which the growing threat of antibiotic-resistant pathogens can be overcome. Herein, we report the potential antimicrobial activity of *Cichorium endivia* L. subsp. *pumilum*. Different concentrations of various solvent extracts prepared from several parts of chicory were tested for their antimicrobial effect against a panel of microorganisms. The antimicrobial activity was analyzed using the well diffusion method, where zones of inhibition were used as indicators of antimicrobial activity. The results indicated the superiority of seed extracts over both leaf and root extracts. Methanol extracts showed higher activity compared with chloroform and water extracts. Increased solvent extract concentration was accompanied by a parallel increase in the diameter of the inhibition zone. Gram-positive bacteria were found to be more sensitive than Gram-negative bacteria and fungi. On a whole, the highest observed inhibition zones (21.3 ± 0.6 and 20.1 ± 0.4 mm) were recorded with the methanolic extract of chicory seeds against *S. aureus* and *B. cereus*, respectively. These results offer insights into the antimicrobial potency of this Egyptian local plant and provide a basis for further phytochemical and pharmacological research.

## 1. Introduction

Since time immemorial, humans and their pathogens have engaged in an everlasting battle. In a distinctive step, human beings discovered antibiotics in the last century. In turn, many microorganisms evolved resistance to antibiotics via natural selection [1]. Nowadays, there is tremendous interest in the extraction of bioactive compounds from natural sources like plants as an alternative to antibiotics. These plant-derived substances have shown reduced instances of side effects and good therapeutic potential. Moreover, several of these proved to have strong antimicrobial activities. Owing to their versatile biological and pharmacological properties, food, nutrition, cosmetics, and pharmaceutical industries have found many applications for these compounds in the production of functional foods, nutritional composites, personal care products, and medicines [2–4].

Although more than 50% of all modern clinical drugs are of natural origin [5], the potential of medicinal plants as a source for new drugs is still highly unexplored. Of the 391,000 plant species currently known to science [6], only a small percentage has been screened for medicinally important compounds. Egypt stands out as a promising source for natural products. The remarkable geographic position of Egypt, along with its varied terrain containing mountains, lakes, deserts, and the longest river in the world, i.e., the Nile river, are responsible for the heterogeneity of its flora. Diverse types of plants grow in the wild in different parts of Egypt. Several medicinal plants have been used to cure specific diseases since ancient times in Egypt; however, most of them have not been scientifically tested for medical use. One of these plants is the Egyptian chicory.

Chicory is a medicinally important plant that belongs to the family Asteraceae. All parts of this plant are pharmacologically useful due to the presence of a number of medicinally important compounds such as alkaloids, flavonoids, inulin, caffeic acid derivatives, sesquiterpene lactones, steroids, terpenoids, oils, volatile compounds, coumarins, and vitamins [7]. It possesses antibacterial [8], antioxidant [9], anti-inflammatory [10], antitumor [11], anti-diabetic [12], and other pharmacological and therapeutic effects. However, the great majority of the published reports worldwide have studied the common chicory (*Cichorium intybus* L.), which is widely grown in Europe, Western Asia, North America, and even in some parts of Egypt. No reports, to our knowledge, have investigated the potential antimicrobial activity of the Egyptian local chicory *Cichorium endivia* subsp*. pumilum*.

*Staphylococcus aureus* is a human pathogen that causes a wide spectrum of diseases, ranging from minor skin infections to fatal necrotizing pneumonia. Although *S. aureus* infections were historically treatable with common antibiotics, the emergence of methicillin-resistant *S. aureus* (MRSA) is now a major concern. MRSA is a multidrug-resistant isolate that is considered the major cause of nosocomial and community-acquired infections [13]. Infections caused by *S. aureus*, above all other antibiotic-resistant strains, have reached epidemic proportions globally [14]. Importantly, some recent studies have reported on the effectiveness of some medicinal plant extracts against MRSA [15–17].

In view of the increasing demand for natural products and the growing threat of multidrug-resistant microorganisms, production of biologically active substances from plant origin is of utmost importance. Considering the reported antimicrobial activity in other species of *Cichorium*, the main aim of the present study was to investigate the antimicrobial potential of different parts of Egyptian chicory, i.e., leaves, roots, and seeds using various aqueous and organic solvents.

## 2. Materials and Methods

### 2.1. Chicory Samples

The seeds of *Cichorium endivia* subsp*. pumilum* were obtained from the Agricultural Research Center, Ministry of Agriculture and Land Reclamation, Egypt. Seeds were immersed in 70% ethanol for 3 min and then rinsed three times in sterile distilled water. Next, the seeds were sterilized for 30 min in 20% commercial Clorox (5% NaOCl) containing 0.5% Tween 20. After rinsing three times with sterile distilled water, aseptic seeds were cultured on MS medium [18]. Leaf and root pieces were harvested from one-month-old seedlings for the preparation of extracts.

### 2.2. Preparation of Extracts

Seeds, leaves, and roots were dried in an oven at 40°C for 24 h. Then, the three dried samples were ground into a fine powder. Methanol, chloroform, and water were used as solvents for the preparation of the extracts. Ten grams of each of the three samples were soaked separately in 100 ml of each solvent and kept in a shaker for 2 d. The obtained mixtures were filtered through Whatman filter paper No. 1. The filtrates were evaporated to near dryness, and the resulting viscous powders were dissolved in the same extract solvents to obtain stock solutions with a final concentration of 50 mg/ml. The stock solutions of the nine extracts were stored at 4°C until use. In this experiment, three test concentrations, i.e., 1.25, 2.5, and 5 mg/ml, obtained from diluting the stock solutions were used.

### 2.3. Microorganisms and Culture Media

The antimicrobial activity of chicory extracts was determined against a panel of pathogens. Two Gram-negative bacteria (*Salmonella typhimurium* NCTC 12023/ATCC 14028 and *Escherichia coli* ATCC 25922), two Gram-positive bacteria (*Bacillus cereus* ATCC 33018 and *Staphylococcus aureus* ATCC 25923), and two fungi (*Candida albicans* CAIM-22 and *Aspergillus niger* ATCC 16404) were provided by the Microbiological Resources Center (MIRCEN), Faculty of Agriculture, Ain Shams University, Egypt. Bacterial strains were maintained on nutrient agar while fungal strains were cultivated on potato dextrose agar (PDA). All cultures were stored at 4°C.

### 2.4. Antimicrobial Assay

Antimicrobial activity tests were conducted by using the agar well diffusion method [19]. Fifteen milliliters of the appropriate agar medium (nutrient agar for bacterial strains and potato dextrose agar for fungal strains) were added into Petri dishes. The melted and tempered (40°C) agar was previously inoculated with 200 *µ*l of the target microorganism cell suspension. The freshly grown suspensions were prepared by diluting microbial cultures of the target strain to achieve a microbial concentration of 10^8^ CFU/ml. The agar plates were solidified for 1 h and then, using a sterile cylinder, wells of 8 mm diameter were made and filled up with 100 *µ*l of the diluted stock solutions (plant extracts). Wells containing pure solvents (100 *µ*l) were used as a negative control, while wells containing standard antimicrobials (chloramphenicol for bacteria and nystatin for fungi) served as a positive control (50 *µ*g/ml). The plates were incubated for 24 h at 37°C and 48 h at 28°C for bacteria and fungi, respectively. The antimicrobial activities of the chicory extracts were evaluated by measuring the inhibition zones around the wells. The inhibition zones were measured with a ruler and were determined by a clear zone of ≥2 mm around the well (diameter of the well: 8 mm). In this investigation, the displayed values represent the inhibition zone diameters excluding the diameter of the wells.

### 2.5. Statistical Analysis

Statistical analysis was performed using IBM SPSS statistics software (base version 25). Factorial analysis of variance (three-way ANOVA) was employed to elucidate the effect of three independent factors on the antimicrobial activity response. The variables used were solvent type (with three values), concentration (with three values), and microorganism type (with six values). Three replicates were used for each treatment. Means and standard errors (SE) were obtained from analysis for each treatment. Data were presented as means ± SE and were compared with Duncan's multiple range test at a 5% probability level.

## 3. Results

The present study demonstrates a comprehensive evaluation of the antimicrobial activity of different solvent extracts prepared from various parts of *Cichorium endivia* subsp*. pumilum* against a broad spectrum of pathogens. In general, irrespective of the solvent type, antimicrobial activity of the chicory seed extract was observed against all microorganisms at varying degrees (Table 1). In this context, methanol extracts showed the highest antimicrobial activity compared to both chloroform and water extracts. It was observed that as the concentration of any solvent extract of chicory seeds increased, the diameter of inhibition zones also increased. Among the different microorganisms, the maximum activity was detected against *S. aureus* (21.3 ± 0.6 mm) followed by *B. cereus* (20.1 ± 0.4 mm), *S. typhimurium* (16.2 ± 0.9 mm), *C. albicans* (14.4 ± 0.7 mm), and *E. coli* (13.7 ± 0.4 mm), while the minimum activity was revealed against *A. niger* (8.3 ± 1.2 mm). All solvent extracts of chicory seeds exhibited stronger activity against Gram-positive bacteria than Gram-negative bacteria and fungi.

Unlike the solvent extracts of chicory seeds, solvent extracts prepared from the leaves of chicory proved to be inefficient against some of the tested microorganisms (Table 2). Regardless of concentration, all the three solvent extracts showed no activity against *A. niger*. Only methanol leaf extract at a high concentration (5 mg/ml) demonstrated inhibitory activity against *E. coli* and *C. albicans,* while neither chloroform extract nor water extract showed any inhibition. At 5 mg/ml, the methanol extract recorded higher activity (9.3 ± 1.5 mm) than the water extract (4.2 ± 1.5 mm) against S. typhimurium, whereas the chloroform extract failed to show any effectiveness. Statistical analysis demonstrated that Gram-positive bacteria (*S. aureus* and *B. cereus*) were the most susceptible microorganisms.

Solvent extracts of chicory roots were less effective as antimicrobial agents compared to solvent extracts prepared from the seeds of chicory. The data in Table 3 indicate the resistance of some microorganisms to solvent extracts of chicory roots. The methanol extract at a low concentration (1.25 mg/ml) affected the growth of only *S. aureus* and *B. cereus*, with inhibition zones of 5.1 ± 1.4 mm and 4.2 ± 1.2 mm, respectively. A higher concentration (5 mg/ml) extended this effect to include *S. typhimurium*, *C. albicans*, and *E. coli*, with inhibition zones of 9.1 ± 1.9 mm, 7.6 ± 2.1 mm, and 4.6 ± 1.5 mm, respectively. No inhibition activities were detected against *A. niger* at all tested concentrations. With respect to chloroform and water extracts, while they showed no effect on the growth of *C. albicans*, *E. coli*, and *A. niger*, they demonstrated antimicrobial activity against *S. aureus*, *B. cereus*, and S. typhimurium. As observed in the case of solvent extracts of chicory seeds and chicory leaves, solvent extracts of chicory roots exerted the largest inhibitory effects, as indicated by the widest inhibition zones, on Gram-positive organisms.

In this study, pure solvents (methanol, chloroform, and water; 100 *µ*l) did not affect the growth of all tested microorganisms. In other words, there was no clear inhibition zone (≥2 mm) recorded around the wells of all negative controls. On the other hand, chloramphenicol (for bacteria) and nystatin (for fungi), which were used as positive controls, showed strong antimicrobial activities against the tested Gram-positive bacteria, Gram-negative bacteria, and fungi with inhibition zones ranging from 20 ± 0.5 to 25 ± 0.6.

## 4. Discussion

Although the results clearly showed that all parts of chicory exhibited antimicrobial activity, solvent extracts prepared from the seeds of chicory exhibited significantly higher activity than those prepared from the leaves and roots of chicory. The varied effects of extracts from different parts of chicory could be attributed to the differences in their phytochemical constituents. Different parts contain different bioactive compounds at different levels, which could have varying effects against microorganisms [20]. This result is consistent with that of Jurgonski et al. [21] who studied the chemical composition of the ethanol extracts of the *Cichorium intybus* seed, peel, leaf, and root and found that the seed extract was the richest source of minerals, fat, protein, and most importantly, phenolic compounds. Recent studies have indicated that phenolics from plant extracts act as antimicrobial agents [22]. They effectively interfere with membrane functions by changing the permeability of cellular membranes, which could lead eventually to the inhibition of microbial growth [23].

Solvent types can also affect the physical properties of the extracts, especially the solubility of phytocomponents. Different solvent extracts have different soluble phytoconstituents in different amounts, and hence, they have varying degrees of antimicrobial activities. In the present investigation, all solvent extracts exhibited antimicrobial activity, but the methanol extracts recorded much higher activity than both chloroform and water extracts. This result suggests that most of the antimicrobial agents in *Cichorium endivia* subsp*. pumilum* are soluble in methanol. Conflicting data have been reported regarding the superiority of methanol extracts over chloroform and water extracts in chicory plants. While some investigators have indicated the superiority of the methanol extract and recommended its use [24], other researchers have declared that aqueous extracts showed the highest antimicrobial activity against some microorganisms [25, 26]. A comparison of the findings of diverse studies is complicated by the fact that there are many factors contributing to differences in the antimicrobial activity of the same solvent. For example, the type of plant material (fresh, frozen, or lyophilized), the mode of extraction (using heat or cold), or the extraction conditions (extraction time, extraction temperature, pH, etc.) may significantly influence the solvent activity [27–30].

In the present study, the increase of solvent extract concentration was accompanied by a parallel increase in the diameter of the inhibition zone. A similar trend has been observed in *Cichorium intybus* extracts [24] as well as in many other medicinal plants such as *Althaea officinalis* [31], *Garcinia mangostana* Linn [32], and *Cassia fistula* Linn [33]. However, the degree of increment differed from one microorganism to another. Thus, the sensitivity to various concentrations of the plant extract depends on the microorganism type. On the other hand, the different concentrations of the solvent extract might explain the varied antimicrobial response of a specific solvent extract prepared from a specific part of a particular plant species against the same pathogen.

Results on the sensitivity of the tested microorganisms to chicory extracts revealed that all tested types of microorganisms were susceptible with different magnitudes. This implies that the mechanism of action of the active principle(s) of chicory extracts is applicable on a broad spectrum of microorganisms. Furthermore, Gram-positive bacterial strains were found to be more sensitive than both Gram-negative bacterial strains and fungal strains. This response might be consistent with the cell wall structure of these microorganisms. While the bacterial cell wall is composed primarily of peptidoglycan [34], the fungal cell wall is composed largely of chitin and other polysaccharides [35]. In addition, Gram-negative bacteria have an extra hydrophilic outer membrane consisting fundamentally of lipopolysaccharides, which inhibit the accumulation of phenolic compounds in the target cell membrane [36]. This renders the Gram-negative bacteria generally more resistant to plant extracts than the Gram-positive bacteria [37]. Our results are in agreement with numerous studies that have reported the antimicrobial effect of *Cichorium intybus* extracts against Gram-positive bacteria, Gram-negative bacteria, yeast, and filamentous fungi [26, 38, 39].

In this work, *S. aureus* was found to be the most susceptible microorganism to *Cichorium endivia* subsp*. pumilum* extracts. Hence, the current finding might be timely and significant, as the seed extracts could be phytochemically screened and further developed into an alternative treatment for MRSA.

## 5. Conclusion

The present study demonstrates a comprehensive evaluation of the antimicrobial activity of different solvent extracts prepared from various parts of *Cichorium endivia* subsp*. pumilum* against a broad spectrum of pathogens. The results indicated the superiority of seed extracts over both leaf and root extracts. The results also indicated the stronger antimicrobial activity of methanol extracts compared with chloroform and water extracts. All tested pathogens were susceptible to chicory extracts; Gram-positive bacterial strains were found to be more sensitive than both Gram-negative bacterial strains and fungal strains. The results of this research provide scientific insights into the antimicrobial potency of this Egyptian local plant and form a basis for further phytochemical and pharmacological research in this field.

## Figures and Tables

**Table 1:** Antimicrobial activity of solvent extracts of *Cichorium endivia* subsp. *pumilum* seeds.

Solvent extract	Concentration (mg/ml)	Zone of inhibition (mm)					
		Gram-positive bacteria		Gram-negative bacteria		Fungi	
		*S. aureus* ^A^	*B. cereus* ^B^	*S. typhimurium* ^C^	*E. coli* ^D^	*C. albicans* ^D^	*A. niger* ^E^
Methanol^a^	1.25	10.4 ± 0.8	7.2 ± 0.7	5.1 ± 0.6	4.2 ± 0.7	4.8 ± 0.5	ND
	2.5	16.7 ± 1.2	15.8 ± 1.1	10.4 ± 0.5	9.1 ± 1.5	8.9 ± 0.8	4.2 ± 0.6
	5	21.3 ± 0.6	20.1 ± 0.4	16.2 ± 0.9	13.7 ± 0.4	14.4 ± 0.7	8.3 ± 1.2

Chloroform^b^	1.25	4.2 ± 0.5	4.1 ± 0.8	ND	ND	ND	ND
	2.5	7.5 ± 0.9	6.8 ± 0.5	4.7 ± 0.6	3.5 ± 0.5	3.7 ± 0.8	ND
	5	12 ± 1.4	11.2 ± 0.9	7.6 ± 0.5	6.6 ± 0.9	6.4 ± 0.7	4.1 ± 0.8

Water^b^	1.25	3.6 ± 1.1	3.7 ± 0.9	3.2 ± 0.7	ND	ND	ND
	2.5	7.1 ± 0.8	6.5 ± 1.3	5.9 ± 1.1	3.1 ± 0.8	3.2 ± 0.9	ND
	5	10.3 ± 1.5	9.9 ± 1.2	9.4 ± 1.3	6.3 ± 1.2	6.1 ± 1.1	3.5 ± 1.5

Chloramphenicol		20 ± 0.5	21 ± 0.4	24 ± 0.5	25 ± 0.6		

Nystatin						24 ± 0.7	24 ± 0.6

Values represent the mean ± standard error (SE) of 3 replicates per treatment. Variable groups that are not represented by the same letters are significantly different (*p* < 0.05). Nystatin (50 *µ*g/ml) and chloramphenicol (50 *µ*g/ml) served as positive controls for fungi and bacteria, respectively. ND: not detected.

**Table 2:** Antimicrobial activity of solvent extracts of *Cichorium endivia* subsp. *pumilum* leaves.

Solvent extract	Concentration (mg/ml)	Zone of inhibition (mm)					
		Gram-positive bacteria		Gram-negative bacteria		Fungi	
		*S. aureus* ^A^	*B. cereus* ^A^	*S. typhimurium* ^B^	*E. coli* ^C^	*C. albicans* ^C^	*A. niger* ^C^
Methanol^a^	1.25	4.8 ± 1.6	3.9 ± 1.4	ND	ND	ND	ND
	2.5	8.9 ± 1.4	8.2 ± 1.7	4.6 ± 1.7	ND	ND	ND
	5	13.2 ± 1.9	14.1 ± 1.8	9.3 ± 1.5	4.1 ± 1.6	5.3 ± 1.9	ND

Chloroform^b^	1.25	ND	ND	ND	ND	ND	ND
	2.5	5.1 ± 1.6	4.8 ± 1.7	ND	ND	ND	ND
	5	9.2 ± 1.9	8.9 ± 1.4	ND	ND	ND	ND

Water^b^	1.25	ND	3.4 ± 1.4	ND	ND	ND	ND
	2.5	4.9 ± 1.8	5.7 ± 1.8	ND	ND	ND	ND
	5	8.6 ± 1.6	10.2 ± 1.7	4.2 ± 1.5	ND	ND	ND

Chloramphenicol		20 ± 0.5	21 ± 0.4	24 ± 0.5	25 ± 0.6		

Nystatin						24 ± 0.7	24 ± 0.6

Values represent the mean ± standard error (SE) of 3 replicates per treatment. Variable groups that are not represented by the same letters are significantly different (*p* < 0.05). Nystatin (50 *µ*g/ml) and chloramphenicol (50 *µ*g/ml) served as positive controls for fungi and bacteria, respectively. ND: not detected.

**Table 3:** Antimicrobial activity of solvent extracts of *Cichorium endivia* subsp. *pumilum* roots.

Solvent extract	Concentration (mg/ml)	Zone of inhibition (mm)					
		Gram-positive bacteria		Gram-negative bacteria		Fungi	
		*S. aureus* ^A^	*B. cereus* ^A^	*S. typhimurium* ^B^	*E. coli* ^CD^	*C. albicans* ^C^	*A. niger* ^D^
Methanol^a^	1.25	5.1 ± 1.4	4.2 ± 1.2	ND	ND	ND	ND
	2.5	9.3 ± 1.7	7.9 ± 1.3	5.4 ± 1.6	ND	3.4 ± 1.3	ND
	5	14.5 ± 1.6	12.6 ± 1.8	9.1 ± 1.9	4.6 ± 1.5	7.6 ± 2.1	ND

Chloroform^b^	1.25	ND	ND	ND	ND	ND	ND
	2.5	4.9 ± 1.5	5.3 ± 1.8	ND	ND	ND	ND
	5	9.8 ± 1.7	9.1 ± 1.5	5.3 ± 1.2	ND	ND	ND

Water^b^	1.25	ND	ND	ND	ND	ND	ND
	2.5	4.5 ± 1.4	5.1 ± 1.8	ND	ND	ND	ND
	5	9.2 ± 1.3	8.8 ± 1.4	3.6 ± 1.6	ND	ND	ND

Chloramphenicol		20 ± 0.5	21 ± 0.4	24 ± 0.5	25 ± 0.6		

Nystatin						24 ± 0.7	24 ± 0.6

Values represent the mean ± standard error (SE) of 3 replicates per treatment. Variable groups that are not represented by the same letters are significantly different (*p* < 0.05). Nystatin (50 *µ*g/ml) and chloramphenicol (50 *µ*g/ml) served as positive controls for fungi and bacteria, respectively. ND: not detected.

## Data Availability

All data generated or analyzed during this study are included within the article.
